# The interlinked trajectories of interpersonal attachment and maladaptive behaviors among Chinese adolescents—a latent growth modeling approach

**DOI:** 10.1186/s13034-025-00943-z

**Published:** 2025-07-22

**Authors:** He Xiao, Pei Chen, Huiyi Xiao, Peizhi Zhong, Jiajie He, Yangang Nie

**Affiliations:** 1 Department of Psychology, Research Center of Adolescent Psychology and Behavior, School of Education, Guangzhou University, Guangzhou, 510006 Guangdong China; 2 Center for Studies of Psychological Application, South China Normal University, Guangzhou, 510631 Guangdong China

**Keywords:** Interpersonal attachment, Maladjustment, Developmental system theories, Latent growth modeling, Adolescent development

## Abstract

**Background:**

In the recent years, global and regional adversities (e.g., strict COVID-19 restrictions, prolonged social isolation, rising academic stress, and increasing digital dependence) have placed youth at heightened risk for ongoing interpersonal, behavioral, and mental health challenges. Examining the relevant trends during this period could yield insight into the persistence of these difficulties and inform targeted intervention strategies. Guided by the theoretical models including developmental system theories and developmental cascade model, the present study focused on three forms of attachment (i.e., child-father attachment, child-mother attachment, and peer attachment) and four types of maladaptive behaviors (i.e., depression, social anxiety, problematic internet use, and academic procrastination) among Chinese adolescents. It aims to uncover their trajectories spanning 2021 to 2023 and the correlations between the trajectories.

**Methods:**

The research draws on data collected at three-time points (i.e., November 2021, May 2022, and May 2023), with a sample of 701 Chinese adolescents (Mage = 14.0 years, SD = 1.44, Range = 13–17 years; 52% girls). A latent growth modeling approach was employed. Specifically, latent growth curve modeling was conducted to estimate the trajectories of the seven key constructs, with Wald tests assessing differences in growth factors across constructs. Slope correlations between attachment and maladaptive behaviors were examined, and Fisher’s z-transformation was applied to compare the strength of these slope-level associations.

**Results:**

(1) All three types of attachment showed declining trajectories, with their slopes positively correlated. (2) Child-father and child-mother attachment declined more steeply than peer attachment. (3) All four maladaptive behaviors demonstrated increasing trends, with their slopes positively correlated. (4) Problematic internet use increased more rapidly than depression and academic procrastination, and social anxiety rose faster than academic procrastination. (5) The slopes of all three types of attachment were negatively correlated with the slopes of all four types of maladaptive behaviors. Notably, child-father and child-mother attachment demonstrated stronger associations with maladjustment trajectories than peer attachment.

**Conclusions:**

In addition to providing longitudinal evidence that adolescents’ interpersonal relationships and maladjustment have been worsening during a time of instability, the study highlights the pivotal role of parent-child relationships and the dynamic interplay between the trajectories of adolescents’ social relationships and behavioral problems. Interventions may need to leverage these trajectory-related characteristics to better enhance adolescents’ psychosocial functioning.

## Background

In the context of ongoing global challenges such as climate change, economic instability, health crises, armed conflicts, and rapid technological advancements, the well-being of children and adolescents remains a pressing concern. Emerging literature [1–4] has documented a troubling decline in mental health outcomes among young populations, with maladjustment manifesting across behavioral, psychological, social, and academic domains [5, 6]. Despite this growing body of evidence, a critical gap lies in understanding how these issues evolved over the last several years, particularly in response to volatile social, economic, and environmental conditions. Notable events included the COVID-19 pandemic (2019–2023), prolonged social isolation (2021–2023), fluctuating inflation rate (peaked in 2022), and the Ukraine-Russia war (2022 to present). These global stressors have coincided with the fluctuations in the stability, functioning, and structure of adolescents’ social environments encompassing family, school, and community [7–9].

Within this global landscape of precarity, Chinese adolescents faced predicaments specific to the sociocultural milieu where they lived. The economic strain placed mounting pressure on families, heightening parental anxieties about their children’s academic performance and prospects, which intensified parent-child conflicts [61]. Strict COVID-19 restrictions and prolonged social isolation hindered peer interactions [62]. As schools reopened, many students struggled with academic challenges such as school-weariness, learning difficulties, and academic procrastination [24]. Digital dependence, while emerging as a coping mechanism initially, developed into internet addiction that extends into the post-pandemic phase [6]. Approximately 15% of Chinese adolescents reported rising levels of mental health difficulties during the COVID-19 pandemic [63]. Against this backdrop, the present study focuses on interpersonal attachment and maladaptive behaviors among Chinese adolescents and aims to explore their trajectories from 2021 to 2023. Recognizing patterns of longitudinal changes in these domains and their interlinks could further the understanding of adolescent adaptation over time, informing strategies to strengthen adolescents’ developmental outcomes in an era of sustained challenges.

### The interpersonal attachment of adolescents

Ecological systems theory deems proximal processes as the primary drivers of development, emphasizing the salience of interactions within the microsystems, such as those with parents, relatives, peers, and teachers [11]. Given the centrality of social relationships in affecting developmental outcomes, this study centers on interpersonal attachment. Inherently relational [12], interpersonal attachment can be conceptualized as the enduring psychological connectedness between individuals, involving critical relational elements such as trust, support, and understanding [13, 14].

Developmental systems theories [11, 15, 16] posit that human development materializes within a dynamic and multi-layered system of interlinked components [17]. While adolescents’ relationships with each significant figure represent distinct subsystems within this broader social ecology, no single bond suffices to fully encapsulate the complexity of adolescents’ proximal social environment. Instead, it is the collective interplay of multiple social relationships that undergird and constitute an individual’s social network [10]. Endorsing this principle, this study examines the attachment with mother, father, and peers, who typically represent the most influential social figures for adolescents.

Attachments first develop through interactions between children and their parents. The significance of a secure child-parent attachment for positive youth development is extensively evidenced [12, 32, 34]. Research further reports the differential contribution of paternal and maternal attachment to adolescent development. In Chinese families, mothers are traditionally more engaged in parenting than fathers, which contributes to stronger emotional bonds between mothers and children [64, 65]. Yet, growing evidence suggests the equal importance of paternal involvement in the socialization of adolescents [66, 67]. As individuals’ social network expands when transitioning to adolescence, peers can emerge as attachment figures comparable to parents. In this study, peer attachment refers to adolescents’ relationships with friends in general, rather than specific close friendships or peer groups. Some research [59] found that peer attachment may exert greater influence than parent-child relationships, while others [9, 68] contend that peer attachment complements rather than replaces the role of parental attachment.

Over the past three years, studies have reported mixed findings regarding changes in adolescents’ social relationships. Decrements in parental support, child–parent attachment, and peer attachment have been documented [8, 9], whereas improvements in child–parent bonds and the maintenance of peer interaction have also been reported [18–20]. Nevertheless, these studies methodologically relied on short-term observations, leaving gaps in capturing the continuity and trajectories of these relational changes. Hence, the current study investigates the trends of child-father, child-mother, and peer attachments over time. Beyond this, the differences in their growth rates are also examined as this may reveal which type of attachment is more stable or steep and suggests which relational domains require more pressing intervention. Accordingly, the study proposes the following research questions:

1. What are the trajectories (i.e., intercept and slope) of the three types of interpersonal attachment?
2. Are the growth rates (i.e., slope) of the three types of interpersonal attachment significantly different?

According to attachment theory [12], the internal working model, constructed through child-parent interactions, is carried into later social interactions and shapes expectations, behaviors, and emotional responses in interpersonal connections. In this sense, child-parent attachment could serve as the prototype for subsequently developed social relationships [10]. Building on the notion of reciprocal influences between subsystems proposed by developmental system theories [11, 15, 16], reciprocity is not limited to family relationships, as seen between child-father and child-mother attachments; it also extends beyond the family system, with child-parent attachment influencing other interpersonal attachments, such as peer attachment, and vice versa [84]. Despite evidence of correlations among child-father, child-mother attachment, and peer attachment in adolescents [21, 22], it remains unclear whether the interrelatedness applies to their trajectories, which hinders the understanding of the extent to which significant interpersonal attachments co-evolve. Thus, the study poses the research question as follows:

3) Are the trajectories of three types of interpersonal attachment (i.e., child-father, child-mother, and peer attachment) correlated over time?

### Maladaptive behaviors of adolescents

The developmental cascade model [23] posits that problematic behaviors in one domain could spill over and generate issues in others, highlighting the interconnected nature of maladaptive behaviors. It is uncommon for adolescents to have struggles in one domain while maintaining good functioning in other areas. More typical scenarios involve adolescents experiencing multiple interrelated difficulties across domains. In this sense, focusing on one type of behavior problem allows for limited knowledge of adolescents’ maladjustment. Hereby, the current research incorporates four related yet distinct types of maladaptive behaviors, i.e., depression, social anxiety, problematic internet use, and academic procrastination, each of which reflects a crucial aspect of adolescents’ maladjustment and has been identified in recent literature as prevalent and impactful among children and adolescents. For example, the prevalence of depression and anxiety have been shown to rise globally in recent years and were strongly associated with social, emotional, and academic difficulties [4]. Academic procrastination has emerged as a significant barrier to learning outcomes and academic achievement [24]. Meanwhile, the increased accessibility of digital devices has amplified concerns around problematic internet use, with excessive screen time being linked with undermined mental health and social disconnection [6]. Furthermore, evidence suggests a high likelihood of co-occurrence and mutual reinforcement among these maladaptive behaviors [25–27].

As opposed to a single type, the inclusion of multiple types of maladaptive behavior may capture a wider spectrum of maladjustment that adolescents wrestled with during the past several years. Akin to the interpersonal relationships, issues remain under-explored around how adolescents’ maladaptive behaviors have evolved, differences in their growth rates, and whether their trajectories were interconnected. Thus, the relevant research questions are proposed:

4) What are the trajectories of four types of maladaptive behaviors (i.e., depression, social anxiety, problematic internet use, and academic procrastination)?
5) Are the growth rates of the four types of maladaptive behaviors significantly different?
6) Are the growth rates of these maladaptive behaviors correlated over time?

### Interpersonal attachment and maladjustment

A secure attachment established in formative years is essential for healthy development throughout the lifespan [13] whereas dysfunctional attachment increases vulnerabilities to developing negative self-concept and behavioral problems [28]. Despite adolescence being a stage marked by self-differentiation and individualization [29], adolescents likely continue to count on external support, approval, and trust to navigate their growingly complex lives featuring social challenges and emotional fluctuations [30]. As such, secure attachments with parents and peers remain vital sources of protective factors for adolescents’ psychosocial development [31–34]. In turn, adolescents’ behaviors and emotional states can influence the quality of their social relationships. Theoretical models, such as ecological systems theory [11] and the transactional model of development [35], emphasize the bidirectional nature of interactions between individuals and their environments. Empirical studies further evidence that maladjustment and interpersonal attachment reciprocally influence one another [36, 37].

Despite the well-documented correlation between interpersonal attachment and maladjustment, much less is known about the interlinks of their developmental trajectories over time. Inquiries such as “When interpersonal attachment quality decreases rapidly, will maladjustment increase rapidly?”, are meaningfully distinct from questions like “When interpersonal attachment quality decreases, will maladjustment increase?”. The former targets the dynamics of change—whether the rate at which individuals experience changes in one domain has relevance to the rate of changes in another. Exploring such trajectory-based associations may uncover the mutuality of developmental processes and inform the design of longitudinal interventions. The current study estimates the correlation between trajectories of depression, social anxiety, problematic internet use, and academic procrastination simultaneously within each attachment domain. This approach methodologically enables detection of both shared and unique co-developmental patterns, controls for overlapping variance among behaviors, and allows for the comparison of relative strengths of correlation across models. Theoretically, it aligns with the transactional perspective emphasizing dynamic developmental processes. In contrast, analyzing each attachment-maladaptive behavior pair separately risks overlooking these interdependencies and inflating Type I error due to multiple testing. Hence, the present study proposes the research question as follows:

7) How are the trajectories (i.e., slopes) of child-father, child-mother, and peer attachment—examined separately—related to the trajectories of depression, social anxiety, problematic internet use, and academic procrastination concurrently?”

### Current study

Guided by multiple theoretical models (i.e., developmental system theories, developmental cascade model), the current study incorporated three types of interpersonal attachment including paternal attachment, maternal attachment, and peer attachment, and four types of maladaptive behaviors encompassing depression, social anxiety, problematic internet use, and academic procrastination. Despite a wealth of empirical evidence highlighting changes in adolescents’ interpersonal relationships, psychosocial functioning, and psychological health in recent years, their trajectories and the links between the trajectories are under-studied. Hereby, the present research draws on three waves of data and employs a latent growth modeling approach to answer the research questions proposed precedingly.

## Method

### Procedure

The current study is part of an extensive longitudinal research project that aims to explore the transactional impacts of gene and family environment on the development of adolescents’ self-control [78]. Employing convenience sampling, the research team recruited students from three middle schools in acity located in southern China. The informed consent of parents and adolescents was collected before the assessment. At the first time point (T1, Nov 2021) of the assessment, research assistants coordinated with the school staff in advance and then visited selected classes to administer the surveys to students. Due to the surge of COVID-19 cases then in China, the assessment at T2 (May 2022) was carried out online and the interval between T2 and T3 was prolonged from six months to one year. At T3 (May 2023), the assessment was restored to be in-person. At the beginning of each assessment, students were thoroughly informed of the purpose, guidelines, and procedure as well as their rights. The assessment took 30–40 minutes. The current study was approved by the institutional review board of the authors’ affiliated university.

### Participants

At T1, 781 students completed the assessment. At T2 and T3, a portion of students missed the assessments due to reasons such as mandatory outreach activities, resulting in 684 and 654 participants completing the full set of surveys at each respective time point. The final sample retained for analysis consisted of 701 students, defined as those who completed most surveys (i.e., missing no more than 10% of the total items) and provided reliable responses, as evidenced by correct answers to lie-detection questions, consistent responses to similar items, and no extended patterns of identical answers. Demographically, the average age of the participants was 14.06 years (SD = 1.44, range = 13–17); boys made up 48% of the sample; a majority of the participants lived in the urban area (*n* = 480, 68.6%); the nuclear family was the predominant family structure (*n* = 632, 90.2%); most of the participants had parents who worked locally (*n* = 615, 87.7%); 69.3% of participants regarded their family economic condition as average. Details on demographics are presented in Table 1.

**Table 1:** Demographic characteristics (*N* = 701)

Variables (*n*, %)						
Age (SD)	14. 06 (1.44)					
Gender	Boy (338, 48.2%)	Girl (363, 51.8%)				
Grade	Grade 7 (452, 64.5%)	Grade 10 (249, 35.5%)				
Family Structure	Nuclear Family (634, 90.4%)	Single-Parent Family (41, 5.8%)	Blended Family (20, 2.8%)	Other (6, 1%)		
Household Location	Country (97, 13.9%)	Town (79, 11.3%)	County (38, 5.4%)	Urban (487, 69.4%)		
Locality of Parents’ Workplace	None of Parents Work Locally (27, 3.8%)	One of Parents Work Locally (84, 8.4%)	Both Parents Work Locally (615, 87.8%)			
Father’s Educational Level	Elementary School or Below (23, 3.3%)	Middle School (176, 25.1%)	High School (229, 25.3%)	College (95, 13.6%)	Bachelor’s Degree (263, 24.2%)	Graduate Degree (59, 8.6%)
Mother’s Educational Level	Elementary School or Below (35, 5%)	Middle School (173, 24.7%)	High School (198, 28.2%)	College* (94, 13.3%)	Bachelor’s Degree (146, 20.8%)	Graduate Degree (56, 8%)
Family Economic Status	Impoverished (5, 0.7%)	Not Wealthy (48, 6.8%)	Average (486, 69.4%)	Somewhat Wealthy (158, 22.5%)	Wealthy (4, 0.6%)	

College In China confers what is analogous to associate degree in higher education system in western countries

### Measures

*Paternal Attachment & Maternal Attachment*. To measure the attachment with parents, the current study adopted an instrument revised by Wang and Song [38] who based upon the parent attachment scale originally constructed by Armsden and Greenberg [31]. The instrument contains anidentical set of 10 items for the father and mother, respectively, which allows for the evaluation of child-parent attachment distinguished by parent gender. This instrument subsumes three dimensions: trust, communication, and alienation. The participants rated each item on a 5-point scale. The final score results from summing up the mean scores of trust and communication and then deducting that of alienation. A high score represents a high level of attachment quality. Items such as “if my father/mother knows something is bothering me, he/she would ask me about it”, and “father/mother accepts the way I am” measure trust and communication while items like “I receive little attention from my father/mother” gauge alienation. In the current sample, the Cronbach’s alphas at T1, T2, and T3 were 0.867, 0.882, and 0.888 for paternal attachment and 0.875, 0.881, and 0.883 for maternal attachment.

*Peer Attachment*. The survey for peer attachment used in the present study also originates from the scale devised by Armsden and Greenberg [31]. The survey is composed of 12 items on three dimensions: trust (e.g., “My friends carefully listen to what I say.”), communication (e.g., “My friends encourage me to speak about my difficulties.”), and alienation (e.g., “Talking about my problems with friends makes me feel embarrassed or stupid.”). The scaling and way of scoring are equivalent to that of the parental attachment instrument. The Cronbach’s alphas of this scale across three time points were α_T1_ = 0.793, α_T2_ = 0.807, and α_T3_ = 0.802.

*Depression*. This externalizing behavior problem was measured using the Center for Epidemiologic Studies Depression Scale (CESD-S) [39]. The instrument comprises 10 items evaluating how frequently a given circumstance happened during the past week, rated on a 4-point Likert scale (i.e., 1 = never/less one day, 4 = almost always/five-seven days). A high score denotes a high level of depressive symptoms. “I struggle with everything” and “I don’t sleep well” are included items. The Cronbach’s alphas for CESD-S in the current sample were 0.816, 0.856, and. 854 across three time points.

*Social Anxiety*. The present study adopted a scale created by Fenigstein et al. [40] to measure social anxiety. The instrument contains six items that are on a 5-point Likert scale. A high score indicates a high level of social anxiety. Example items are “It takes me a good amount of time to overcome shyness in a new environment” and “Being around a large group of people makes me nervous”. The Cronbach’s alphas of the scale in the current sample were 0.685, 0.739, and 0.731.

*Problematic Internet Use*. The scale devised by Young [41] was utilized to measure how problematic adolescents’ internet use was. The scale includes 10 items. Respondents rated each item on how well it reflected the perceived reality, using a scale from 1 = not at all to 6 = absolutely. Some items are “Is it hard for you to reduce or control internet use?” and “Does the time spent on the internet exceed your expectations?”. The Cronbach’s alphas of the social anxiety instrument in the present study were 0.896, 0.907, and 0.912.

*Academic Procrastination*. The current study adopted an instrument [42] that originated from the scale constructed by Lay [43] to measure academic procrastination. The instrument is composed of 19 items, e.g., “I oftentimes postpone the mandatory learning tasks”, and “When the assignment is due, I am doing something irrelevant”. Participants evaluated to what extent each item aligned with their situation on a 5-point Likert scale (i.e., 1 = not at all, 5 = absolutely). The Cronbach’s alphas of the academic procrastination scale in the current sample were 0.905, 0.905, and 0.908.

### Missing data

For cases with extensive missingness (e.g., missing more than 10% of the total items) or unreliable responses, listwise deletion was applied. Although the remaining missing data were not missing completely at random, as indicated by a significant result of Little’s MCAR test (*p* <.05), the missingness in this study was primarily due to external, administrative factors (mandatory outreach programs and schedule conflicts), which are unrelated to the key constructed under investigation. These circumstances render plausible the assumption that the data were missing at random (MAR), and thus multiple imputation (MI) was then employed. In the MI procedure, all seven key study variables (i.e., three attachment variables and four maladjustment variables) were included as both predictors and outcomes to maximize the accuracy of the imputed values. A total of 10 imputed datasets were generated using SPSS 29.0, and analyses were conducted on the pooled results based on Rubin’s rules [55].

### Statistical analysis

The study employed SPSS 29.0 to conduct preliminary analyses. Upon deriving the descriptive statistics, a series of t-tests were performed to compare the participants retained in the final sample with those excluded to assess sample representativeness and potential bias. Then, bivariate correlation was measured in two ways:(1) each variable was correlated with itself across time points, and (2) variables were correlated with one another within each time point. All analyses concerning latent growth modeling were conducted in Mplus 8.3 [44].

The analytic procedure contained multiple steps. First, measurement invariance was assessed individually for the seven key constructs— three types of attachments and four types of maladaptive behaviors. A stepwise procedure was adopted to evaluate configural, metric, and scalar invariance through sequential model comparisons (i.e., configural vs. metric, and metric vs. scalar) [62]. Invariance was determined using commonly used cutoff criteria: ΔRMSEA < 0.015, ΔCFI < 0.010, and ΔSRMR < 0.010 [69]. A construct is considered to meet invariance if at least two out of the three criteria are satisfied. When full scalar invariance could not be established, partial scalar invariance was tested. This involved identifying a potentially misfitting item, releasing its intercept constraint, and comparing the revised model with the metric invariance model using the same set of cutoff criteria. If the model fit remained inadequate, additional item constraints were released until partial scalar invariance was achieved [62]. For constructs that met either full or partial scalar invariance, the linear latent growth modeling was applied to estimate its trajectory reflected by intercept and slope growth factors [70].

Of note, in the current study the time interval between T1 and T2 was six months whereas the lag between T2 and T3 extended to one year due to pandemic-related restrictions. To account for the effects of unequal time intervals when estimating the slope, the growth model syntax was specified as “i s| T1@0 T2@1 T3@3” rather than the typical “i s| T1@0 T2@1 T3@2.” Following the latent growth modeling, Wald test was performed to examine the differences in growth factors among attachment variables and maladaptive behavior variables, respectively. 95% confidence interval (CI) based on 1000 times of bootstrapping were calculated to evaluate the significance of differences. Subsequently, the correlations between the slopes of three types of interpersonal attachment and the slopes of four types of maladaptive behaviors were assessed. These steps responded to research questions #1 through #6.

In the final step, which addressed research question #7, three separate models were created to estimate how the trajectories of each type of interpersonal attachment—child-father, child-mother, and peer attachment—relate to the trajectories of maladjustment. In each model, one type of attachment was paired concurrently with all four maladaptive behavior constructs (i.e., depression, social anxiety, problematic internet use, and academic procrastination).

Within each model, the intercept growth factor of the attachment was set to predict its own slope as well as the slopes of the four maladaptive behaviors, gauging to what extent the initial attachment level was associated with changes in behavioral outcomes over time. More importantly, the slope of the attachment was specified to correlate with the slopes of the maladaptive behaviors, capturing their co-developmental patterns.

To preliminarily compare the strength of these slope-level associations across the three attachment models, Fisher’s z-transformation was applied to calculate 95% CIs of the standardized correlation coefficients between attachment slopes and behavior slopes. The degree of overlap between 95% CIs was used to assess whether the strength of these correlations differed meaningfully across attachment types.

## Results

The t-tests based on T1 data showed that, demographically, the final sample differed from the excluded group on family economic status, family structure, and mother’s educational level, but not on age, gender,, and father’s educational level. Moreover, no significant differences were found across the seven key constructs (i.e., the three types of attachment and four types of maladaptive behaviors). This suggests that the final sample may retain reasonable representativeness despite the unknown influences of the excluded participants. The bivariate correlation analysis showed each variable had at least a moderate correlation with itself from a different time point. Meanwhile, all seven variables were significantly associated with one another at every time point. As expected, three kinds of interpersonal attachment were positively related, and so were the four kinds of maladaptive behaviors. Attachment variables were inversely correlated with maladjustment variables (see detailed output in Table 6 in the supplemental material).

Measurement invariance testing indicated that all three types of attachment met requirements for configural, metric, and scalar invariance. As the attachment constructs subsume three dimensions—communication, trust, and alienation, these dimensions were used as the indicators of latent factors of each time point. The four maladaptive behavior constructs, while their full scalar invariance could not be established, met the criteria for partial scalar invariance. In the model of depression, items #5 and #8 were first deleted due to their low factor loading; and the intercept constraint of item#1 was released. In the model of social anxiety, the intercept constraints of items #1 and #3 were removed. For problematic internet use, the intercept constraints of items #7 and #9 were released. The academic procrastination scale, which comprises 19 unidimensional items, was modeled using five item parcels to balance content coverage, model complexity, and interpretability. Items were randomly assigned to parcels to ensure distributional balance [73]. Partial scalar invariance was established by releasing the intercept constraints for parcels #1, #3, and #5 (see Table 2 for details).

**Table 2:** Measurement invariance

Model fit								Model comparisons				
	Model	χ^2^	df	CFI		RMSEA	SRMR	Models	Δχ^2^	ΔCFI	ΔRMSEA	ΔSRMR
CFA												
	Configural(M1)	42.163	15	0.987		0.051	0.038					
	Metric (M2)	45.781	19	0.988		0.045	0.040	M2 - M1	3.618	0.001	− 0.006	0.002
	Scalar (M3)	55.847	23	0.985		0.045	0.040	M3 - M2	10.066	− 0.003	0	0
CMA												
	Configural(M1)	36.633	15	0.990		0.045	0.027					
	Metric (M2)	40.492	19	0.990		0.040	0.030	M2 - M1	3.859	0	− 0.005	0.003
	Scalar (M3)	49.776	23	0.988		0.041	0.032	M3 - M2	9.284	− 0.002	0.001	0.002
PA												
	Configural(M1)	39.537	18	0.987		0.041	0.049					
	Metric (M2)	41.951	22	0.988		0.036	0.053	M2 - M1	2.414	0.001	− 0.005	0.004
	Scalar (M3)	65.912	28	0.977		0.044	0.059	M3 - M2	23.961	− 0.011	0.008	0.006
DP												
	Configural(M1)	565.124	222	0.944		0.047	0.041					
	Metric (M2)	582.104	236	0.943		0.046	0.043	M2 - M1	16.98	− 0.001	− 0.001	0.002
	Scalar (M3)	684.861	252	0.929		0.050	0.055	M3 - M2	102.757	− 0.014	0.004	0.012
	Partial scalar (M4)	658.605	250	0.933		0.048	0.052	M4 - M2	76.501	− 0.010	0.002	0.009
SA												
	Configural(M1)	388.395	114	0.943		0.059	0.056					
	Metric (M2)	407.534	124	0.941		0.057	0.057	M2 - M1	19.139	− 0.002	− 0.002	0.001
	Scalar (M3)	523.976	136	0.919		0.064	0.070	M3 - M2	116.442	− 0.022	0.007	0.013
	Partial scalar (M4)	464.527	132	0.931		0.060	0.064	M4 - M2	56.993	− 0.010	0.003	0.007
PIU												
	Configural(M1)	830.078	369	0.951		0.042	0.036					
	Metric (M2)	1011.371	387	0.951		0.041	0.039	M2 - M1	181.293	0	− 0.001	0.003
	Scalar (M3)	992.727	407	0.938		0.045	0.053	M3 - M2	-18.644	− 0.013	0.004	0.014
	Partial scalar (M4)	955.580	403	0.941		0.044	0.050	M4 - M2	-55.791	− 0.010	0.003	0.011
AP												
	Configural(M1)	180.604	72	0.984		0.046	0.024					
	Metric (M2)	194.756	80	0.983		0.045	0.029	M2 - M1	14.152	− 0.001	− 0.001	0.005
	Scalar (M3)	322.417	90	0.965		0.061	0.057	M3 - M2	127.661	− 0.018	0.016	0.028
	Partial scalar (M4)	255.190	84	0.974		0.054	0.042	M4 - M2	60.434	− 0.009	0.009	0.013

*CFA* child-father attachment, *CMA* child-mother attachment, *PA* peer attachment, *DP* depression, *SA* social anxiety, *PIU* problematic internet use, *AP* academic procrastination

It warrants noting that in each of the latent growth models for the seven constructs, latent factors at each time point were indicated by either items, dimensions, or parcels, and the latent growth factors (i.e., intercept and slope) were modeled at a higher order. This structure renders the model the second-order latent growth model (also referred to multiple indicator linear growth model). When parameterizing such models in Mplus, the mean of the intercept growth factor is fixed as zero and the mean of the slope growth factor is estimated [44].

As shown in Table 3, the slope growth factors of all variables were significantly different from zero. The significant growth rates indicated that all variables at the intraindividual level changed in a statistically meaningful way across three time points. A pattern emerged that all three types of interpersonal attachment showed a decremental trend whilst all four types of maladaptive behaviors displayed an incremental trend. Furthermore, the pairwise comparison test showcased that the slopes of child-father and child-mother attachment were not significantly different; yet they differed from that of peer attachment, displaying a steeper declining trend. Among the maladaptive behaviors, problematic internet use had a higher increasing rate than depression and academic procrastination. Social anxiety exhibited a higher incremental rate than academic procrastination. No significant differences were observed in other pairwise comparisons.

**Table 3:** Unstandardized growth estimates and their differences

	Slope	Pairwise comparison
CFA	− 0.08***	CFA = CMA 0.003, 95% CI [-0.018, 0.014] CFA < PA − 0.050**, 95% CI [-0.079, -0.022] CMA < PA − 0.047**, 95% CI [-0.074, -0.021]
CMA	− 0.077***	
PA	− 0.022*	
DP	0.068***	SA = DP 0.019, [-0.013, 0.049] SA = PIU − 0.026, [-0.061, 0.005] SA > AP 0.029*, [0.001, 0.058] PIU > DP 0.045**, [0.014, 0.075] PIU > AP 0.054***, [0.032, 0.083] DP = AP 0.009, [-0.032, 0.018]
SA	0.086***	
PIU	0.110***	
AP	0.058***	

In Mplus, the intercept growth factor is fixed to zero by default as part of the parameterization requirements for multiple indicator linear growth models; thus, they are not presented

*CFA* child-father attachment, *CMA* child-mother attachment, *PA* peer attachment, *DP* depression, *SA* social anxiety, *PIU* problematic internet use, *AP* academic procrastination

***p <.001, **p <.01, *p <.05

Correlations among the growth factors within the attachment domain revealed that the intercepts of child-father, child-mother, and peer attachment were positively associated, with the r correlation coefficients spanning 0.252 to 0.510. Similarly, positive correlations among their slopes were observed, though smaller in magnitude (*r* =.016 to 0.050). Within the maladjustment domain, the initial levels of the four maladaptive behaviors were also positively correlated, with *r* ranging from 0.110 to 0.384. In parallel, their growth rates showed significantly, albeit limited, positive associations, with *r* falling between 0.007 and 0.022 (see Table 4).

**Table 4:** Unstandardized correlations coefficient between growth factors

Interpersonal attachment												
			Intercept correlation						Slope correlation			
			1		2				1		2	
1. CFA												
2. CMA			0.51***						0.05***			
3. PA			0.252***		0.256***				0.017 ***		0.016***	
Maladaptive behaviors												
	Intercept correlation							Slope correlation				
		1		2		3	1			2		3
1. DP												
2. SA		0.272***					0.016***					
3. PIU		0.267***		0.384***			0.021***			0.016**		
4. AP		0.11***		0.18***		0.29***	0.007***			0.009***		0.022***

*CFA* child-father attachment, *CMA* child-mother attachment, *PA* peer attachment, *DP* depression, *SA* social anxiety, *PIU* problematic internet use, *AP* academic procrastination

***p <.001, **p <.01, *p <.05

The three latent growth models, each of which paired one type of interpersonal attachment concurrently with four types of maladaptive behaviors, yielded an array of statistically significant results (Table 5). In the child-father attachment model (χ^2^(4424) = 8014.228, *p* <.001, RMSEA = 0.034, CFI = 0.916, TLI = 0.913, SRMR = 0.052), the intercept of the attachment negatively predicted its own slope (*B*=-0.082) and positively predicted the slopes of all maladaptive behaviors (*B* = 0.025 to 0.082). Additionally, the slope of the child-father attachment was negatively associated with the slopes of four types of maladaptive behaviors, with *r* spanning − 0.009 to − 0.017. The child-mother attachment model (χ^2^(4424) = 8054.279, *p* <.001, RMSEA = 0.034, CFI = 0.915, TLI = 0.912, SRMR = 0.053) produced results comparable to the child-father attachment model. The attachment intercept negatively predicted its own slope (*B*=-0.07) and positively predicted the slopes of maladaptive behaviors (*B* = 0.036 to 0.098). The attachment slope had a negative association with the slopes of four types of maladaptive behaviors (*r=*-.009 to − 0.017). In the peer attachment model (χ^2^(4425) = 8426.351, *p* <.001, RMSEA = 0.036, CFI = 0.905, TLI = 0.902, SRMR = 0.070), the attachment intercept negatively predicted its own slope (*B*=–0.094) and positively predicted the slopes of all maladaptive behaviors except depression, with *r* ranging from 0.046 to 0.121. However, the peer attachment slope was only significantly associated with the slope of academic procrastination (*r* =–.005); slope-slope correlations with other maladaptive behaviors were not significant.

**Table 5:** Three attachment-maladjustment growth factors models

	Intercept predicts slope	B	Slope-slope association	*r*	*r*_s_	95% CI	Relative strength of slope-slope association
Father-Child Attachment Model	S_F_ on I_F_ S_A_ on I_F_ S_D_ on I_F_ S_P_ on I_F_ S_X_ on I_F_	− 0.082*** 0.025* 0.062*** 0.077*** 0.082***	S_F_ w S_A_ S_F_ w S_D_ S_F_ w S_P_ S_F_ w S_X_	− 0.009*** − 0.013*** − 0.017*** − 0.011**	− 0.459 − 0.758 − 0.415 − 0.327	[-0.519, − 0.393] [-0.787, − 0.724] [-0.478, − 0.347] [-0.398, − 0.254]	Depression Father-Child = Mother -Child > Peer Attachment Problematic Internet Use Father-Child = Mother-Child > Peer Attachment Social Anxiety Father-Child = Mother- Child > Peer Attachment Academic Procrastination Father-Child = Mother-Child = Peer Attachment
Mother-Child Attachment Model	S_M_ on I_M_ S_A_ on I_M_ S_D_ on I_M_ S_P_ on I_M_ S_X_ on I_M_	− 0.070** 0.036** 0.054** 0.098*** 0.079***	S_M_ w S_A_ S_M_ w S_D_ S_M_ w S_P_ S_M_ w S_X_	− 0.009*** − 0.013*** − 0.017*** − 0.009**	− 0.512 − 0.801 − 0.450 − 0.265	[-0.568, − 0.451] [-0.825, − 0.772] [-0.510, − 0.384] [-0.336, − 0.192]	
Peer Attachment Model	S_E_ on I_E_ S_A_ on I_E_ S_D_ on I_E_ S_P_ on I_E_ S_X_ on I_E_	− 0.094*** 0.046* 0.041 0.121** 0.094*	S_E_ w S_A_ S_E_ w S_D_ S_E_ w S_P_ S_E_ w S_X_	− 0.005** − 0.001 − 0.006 − 0.001	− 0.396 − 0.050 − 0.252 − 0.017	[-0.459, − 0.328] [-0.123, 0.024] [-0.326, − 0.176] [-0.091, 0.057]	

*I* intercept, *S* slope, *F* father-child attachment, *M* mother-child attachment, *E* peer attachment, *A* academic procrastination, *D* depression, *P* problematic internet use, *X* social anxiety. For example, *I*_*F*_ intercept of father-child attachment, *S*_*D*_ slope of depression. *on* regressed on, *w* correlate, *r* unstandardized correlation coefficient, *r*_*s*_ standardized correlation coefficient

***p <.001, **p <.01, *p <.05

Examination of the 95% confidence intervals of standardized slope-slope correlations indicated no significant differences between child-father and child-mother attachment, as their intervals overlapped across all maladaptive behaviors. In contrast, correlations involving peer attachment were significantly weaker for depression, problematic internet use, and social anxiety than child-father and child-mother attachment, as reflected by non-overlapping intervals (see the final two columns in Table 5 ).

**Table 6:** Bivariate correlation within and between variables

	Mean (SD)						Correlation within variables						
	T1		T2		T3		T1—T2			T1—T3		T2—T3	
CFA	1.29(0.82)		1.13(0.86)		1.06(0.85)		0.653**			0.554**		0.626**	
CMA	1.45(0.79)		1.28(0.83)		1.23(0.81)		0.662**			0.560**		0.660**	
PA	1.64(0.60)		1.57(0.62)		1.53(0.59)		0.577**			0.473**		0.508**	
DP	1.83(0.71)		1.99(0.61)		2.09(0.60)		0.668**			0.390**		0.622**	
SA	2.95(0.84)		3.12(0.84)		3.26(0.79)		0.554**			0.432**		0.546**	
PIU	2.65(1.06)		2.94(1.08)		3.08(1.10)		0.598**			0.531**		0.593**	
AP	2.35(0.71)		2.51(0.70)		2.61(0.71)		0.682**			0.596**		0.682**	

Correlation between variables													
		1		2		3		4	5		6		
T1													
1. CFA													
2. CMA		0.806**											
3. PA		0.379**		0.410**									
4. DP		− 0.375**		− 0.366**		− 0.340**							
5. SA		− 0.279**		− 0.259**		− 0.281**		0.341**					
6. PIU		− 0.358		− 0.333**		− 0.307**		0.409**	0.418**				
7. AP		− 0.400**		− 0.387**		− 0.299**		0.317**	0.355**		0.563**		
T2													
1. CFA													
2. CMA		0.817**											
3. PA		0.366**		0.388**									
4. DP		− 0.463**		− 0.450**		− 0.342**							
5. SA		− 0.172**		− 0.121**		− 0.154**		0.356**					
6. PIU		− 0.292**		− 0.256**		− 0.258**		0.380**	0.263**				
7. AP		− 0.345**		− 0.356**		− 0.279**		0.389**	0.211**		0.526**		
T3													
1. CFA													
2. CMA		0.800**											
3. PA		0.330**		0.330**									
4. DP		− 0.431**		− 0.426**		− 0.317**							
5. SA		− 0.138**		− 0.117**		− 0.184**		0.330**					
6. PIU		− 0.284**		− 0.280**		− 0.201**		0.453**	0.235**				
7. AP		− 0.354**		− 0.361**		− 0.266**		0.438**	0.197**		0.596**		

*CFA* child-father attachment, *CMA* child-mother attachment, *PA* peer attachment, *DP* depression, *SA* social anxiety, *PIU* problematic internet use, *AP* academic procrastination

**p <.01

## Discussion

The present study employed the latent growth modeling approach to probe the trajectories of interpersonal attachment and maladjustment among Chinese adolescents from 2021 to 2023. Beyond identifying the trends, the study further assessed the differences of growth rates, the associations between these trajectories, and the relative strength of these associations. The discussion that follows is structured by the research questions.

### The interlinked downward trends of interpersonal attachment (RQ#1, RQ#2, RQ#3)

The study uncovered a declining trajectory in child-father attachment, child-mother attachment, and peer attachment, respectively, which reflected a downward trend of overall interpersonal attachment quality. This pattern suggests that between 2021 and 2023, adolescents may have received less social support in the forms of trust and communication, and endured a heightened feeling of alienation from their social environment. The widespread and prolonged impact of public health crises, economic strain, and sociocultural shifts during this period led to systemic disruptions in adolescents’ relational contexts involving family dynamics and peer interaction [2, 4]. These persistent and multi-level stressors spanning individual, familial, community, and societal domains, likely contributed to the concurrent deterioration in the quality of adolescents’ multiple interpersonal attachments.

As opposed to the prior studies [8, 9, 18–20] that reported changes in social relationships during the past several years, the current study extends the literature by contrasting the rates of change across interpersonal attachments. While the decremental rates in child-father and child-mother attachment were statistically indistinguishable, they both displayed much steeper declines than peer attachment.

The normative developmental shifts in parent-child relationships during adolescence are characterized by decreased communication, reduced closeness, and increased conflict [79, 80]. Under typical circumstances, these changes do not necessarily undermine attachment security [81]. However, during a period of adversity and instability (e.g., the COVID-19 pandemic), the impacts of these developmental patterns may be amplified. Parents faced compounded stressors such as financial insecurity, the competing demands of work and caregiving, illness or loss of family members, reduced community support [7, 18, 19]. These strains may result in increased burnout, anxiety, and emotional distress, potentially compromising parental involvement and practices [45]. Diminished emotional availability and heightened stress likely weakened the quality of parent-child relationships and elevated the risk of maladaptive parenting behaviors [46–48].

In contrast, adolescent peer relationships typically undergo developmental gains marked by increased integration, self-disclosure, intimate communication, and emotional support [82, 83]. Although disruptions to school routines and limited in-person contact during the pandemic intensified adolescents’ loneliness and hindered the development of social competencies [49, 50], the eventual reopening of schools provided opportunities to restore these peer-oriented developmental benefits. This could serve as a buffer, rendering peer relationships a more adaptive and flexible relational domain during this period, despite ongoing challenges in social reintegration [51]. Taken together, it is inferred that systemic stressors, when combined with normative developmental changes, may have impacted family-based attachments more acutely than peer relationships, contributing to the sharper decline observed in child-parent attachment relative to peer attachment.

The trends of three types of interpersonal attachments were found to be positively correlated. In line with the developmental system theories [11, 15, 16] that highlight the dynamic and reciprocal interactions of developmental domains, the current study demonstrates that in the cases where the quality of one type of attachment declines rapidly, the quality of other types of attachment is likely to drop at a high rate. Conversely, when the quality of attachment with one significant figure increases more quickly, attachments with other figures may improve more rapidly. Such dynamics hint at the possibility that adolescents’ different social relationships may co-develop in a mutually enhancing fashion.

### The interlinked upward trends of maladaptive behaviors (RQ#4, RQ5, #RQ6)

In contrast to interpersonal attachment, all four types of maladaptive behaviors (i.e., depression, social anxiety, problematic internet use, academic procrastination) exhibited an upward trajectory. In line with the prior studies [4, 51], the current research showcased that adolescents’ maladjustment has not abated over time; instead, multiple maladaptive behaviors have persisted and even intensified.

Research has revealed divergent developmental courses of maladjustment among adolescents. Most youth tend to follow a normative path in which they show consistent and low levels of behavioral problems [85, 86]. A subset demonstrates diminishing levels of conduct problems and emotional disturbances over time, possibly due to the natural resolution of early difficulties during the transition from childhood to adolescence [85–87]. However, the emergence or accumulation of risk factors may disrupt this stability or impede recovery. Essler et al. [52] found fluctuations in psychological health with the improvement after the first lockdown in the spring of 2020, followed by declines during the second lockdown in the winter of 2020. A meta-analysis by Miao et al. [53] identified temporal variations in mental health issues, such as anxiety and depressive symptoms, reflecting the dynamic nature of these problems. The exposure to early adversity [87] and declining perceived school climate [88] have also been linked to the increasing trend of problematic behaviors. These findings suggest that the upward trends observed in the four maladaptive behaviors may be partially attributable to a combination of deteriorating interpersonal attachment quality and the buildup of environmental stressors in the past several years.

Going beyond prior studies that have examined the changes in maladjustment indicators among Chinese youth [6, 71, 72], the present study compared the change rate of multiple maladaptive behaviors within a longitudinal framework. While a few significant differences were identified, such as problematic internet use increased more rapidly than depression and academic procrastination, and social anxiety grew faster than academic procrastination, the overall results do not form a clear hierarchy.

Problematic internet use has been linked to emotional dysregulation [74]. The escalating mental distress experienced from 2021 to 2023 may have led adolescents to rely more heavily on digital engagement, thereby accelerating its growth relative to other behaviors. Similarly, social anxiety has a bidirectional relationship with friendship quality, peer rejection, and victimization [75]. Amid changing social norms and uncertainty in peer contact during this period, socially anxious adolescents may have faced greater struggles of sustaining connections. This cycle of precarious peer experiences and rising anxiety may have contributed to the faster growth rate of social anxiety. These tentative inferences underscore the possibility that maladaptive behaviors do not evolve uniformly, but follow distinct yet occasionally parallel developmental courses, which warrants future research into the mechanisms driving variations in these trajectories.

The positively associated trends of the four types of maladaptive behaviors showcase that a worsening trend in one behavior problem may be accompanied by parallel declines in others. This finding resonates with the developmental cascade model [23], suggesting that behavior problems are typically not isolated occurrences but are interlinked facets of a broader maladjustment process. These interrelated maladaptive behaviors may develop into a self-perpetuating cycle, where issues in one domain spill over to trigger and compound problems in other areas.

### The interlink between interpersonal adjustment and maladjustment (RQ#7)

The current study found that the initial level of interpersonal attachment negatively predicted its own developmental trajectory, indicating that adolescents who began with higher-quality attachment to fathers, mothers, or peers experienced a steeper decline over time. This may reflect a regression to the mean or a normative developmental shift, where adolescents with initially strong attachments have more room to decrease, or they gradually move toward higher levels of autonomy and self-differentiation [29]. Besides, higher initial levels of all three types of attachment were positively associated with the growth of maladaptive behaviors. This somewhat counterintuitive finding may suggest that the effects of early high attachment weakened over time, or were insufficient to help buffer against the accumulating stressors adolescents faced between 2021 and 2023.

The negative correlations between the slopes reveal that adolescents who experienced a more rapid increase in maladaptive behaviors tended to demonstrate either slower growth or faster decline in their attachment quality with parents or peers. Conversely, adolescents whose attachment strengthened more rapidly could experience a deceleration in maladaptive behaviors. These findings align with prior research [20, 34, 54] that highlighted the protective effect of interpersonal attachment on adolescents’ health outcomes. Literature has established that adolescents’ social relationships and behavior problems act as both precursors and consequences of each other [36, 37]. However, the findings are mainly concerned with “static” relationships—addressing how changes in one variable relate to concurrent or subsequent shifts in another. Yet, development could unfold in the form of a trajectory, be it linear, curvilinear, or otherwise. Probing correlations between rates of change allows for a dynamic understanding of how developmental domains co-evolve. The observed negatively correlated trends between interpersonal attachment and maladaptive behaviors in the current study add a longitudinal dimension to their transactions, suggesting that these two developmental dimensions may mutually shape each other’s trajectories.

In the current study, child-father and child-mother attachment were found to have modest associations of similar strength with the trajectories of adolescent maladaptive behaviors, while peer attachment demonstrated consistently weaker and mostly non-significant associations. Although prior research has emphasized the primacy of maternal attachment in predicting adolescent adjustment [76], emerging evidence points to the equal significance of paternal involvement in the socialization of children [67]. Through adolescence, the weight of paternal role may increase whilst maternal impact could diminish [77]. This shift is particularly relevant in the Chinese context, where mothers have traditionally been more involved in caregiving [64], but nowadays fathers are increasingly engaged as parenting responsibilities become more equally shared in the context of maternal employment.

Consistent with prior findings, peer attachment appears to supplement rather than replace parental bonds during adolescence [68]. Although its correlations were weaker, peer attachment remains relevant for understanding (mal)adjustment trajectories as peer relationships, marked by greater symmetry than parent-child ties, offer unique developmental functions such as identity exploration [10].

## Implications

Interventions aimed at restoring the psychosocial functioning of youth following difficult times are oftentimes more effective when adopting a comprehensive approach that involves multiple subsystems and encourages multi-level collaboration [10, 56]. However, in practice, implementing such all-encompassing strategies is pragmatically difficult and resource-intensive. A nuanced and complementary approach could be targeting a single, high-impact social relationship that presents the greatest leverage for change. Predicated on the current study’s findings, child-parent attachment may warrant greater initial attention. Moreover, child-parent attachment may be more amenable to change through family-based efforts. In contrast, enhancing peer attachment may require broader school-wise engagement, which poses pragmatic challenges due to time constraints and a lack of specialized training among educators—a prevalent situation in Chinese high schools. While peer relationships remain important, the practical and empirical rationale supports prioritizing parent-child bonds as a starting point. Meanwhile, intervention strategies need to be flexible, allowing room for adjustment to accommodate the unique needs and social contexts of adolescents.

The interconnected nature of maladaptive behaviors entails interventions to consider and address multiple issues concurrently, rather than separating them. In theory, resources should be geared toward the problems that are most impactful and serious. However, identifying the most pressing issues is difficult as it may vary across contexts and time. Instead of singling out one behavior for focused intervention, a more effective approach may be implementing programs that target shared underlying and modifiable mechanisms, such as emotion regulation deficits and maladaptive stress coping strategies. From a preventive standpoint, it remains essential for parents, teachers, and other caregivers to stay vigilant to early signs of problematic behaviors and intervene proactively to prevent their deterioration.

## Limitation

Attention needs to be paid to several limitations of the study. While the current study identifies the trends of interpersonal attachment and maladjustment of adolescents from 2021 to 2023, it did not examine the specific drivers or mechanisms underlying these trajectories. Further inquiries are necessary to determine the explicit forces—individual, relational, and contextual—that shape adolescent health trajectories. Although the model fit indices for the three latent growth models were within acceptable ranges (e.g., RMSEA = 0.034–0.036; CFI = 0.905–0.916; SRMR = 0.052–0.070), they did not reach the conventional thresholds for excellent fit (e.g., CFI/TLI ≥ 0.95). This may be due to the complexity of the models, which contained numerous item-level indicators and partial scalar invariance. Relatedly, the study did not include demographic covariates such as age, gender, or family socioeconomic status in the models in order to manage the complexity and interpretability of the analyses. Previous research [57, 58] suggests that girls, higher-graders, and those from lower socioeconomic backgrounds are more vulnerable to experiencing maladjustment during adolescence. Hereby, the less-than-optimal model fit and the omission of these covariates could limit the precision of parameterization and should be considered when interpreting the findings.

Besides, the sample was drawn from adolescents attending schools in a single city in China. As such, the findings may not be fully representative of the broader adolescent population across diverse regions in China or other cultural contexts. This points to the need for replication using more diverse and nationally representative samples. Additionally, the study did not account for other salient social relationships, such as the student–teacher relationship, which has been shown to play a critical role in adolescent development [60]. Future research may need to carefully consider what type of interpersonal relationships to factor in and how to organize them to accurately mirror the configuration, content, and dynamics of adolescents’ broader social ecological systems. Ultimately, the four types of maladaptive behaviors under investigation primarily reflect the adolescents’ dysfunction. Future research would benefit from taking a positive psychology perspective by incorporating constructs such as emotional resilience, life satisfaction, gratitude, and prosocial behaviors to develop a more holistic understanding of adolescent development.

## Conclusion

The present study revealed that from 2021 to 2023, a period marked by persistent strains and stressors in adolescents’ ecological environments, a group of Chinese (and possibly many youths globally) experienced worsening maladaptive behaviors while navigating a social landscape characterized by diminishing support and increasing disconnection. Of note, the trajectories of interpersonal attachments and maladjustment were found to be interlinked, which adds a longitudinal lens through which to make sense of their interplay. This understanding could help inform collective and strategic efforts aimed at transforming current challenges into pathways for enhancing adolescent well-being in an increasingly complex and demanding world.

## Data Availability

The datasets generated during and analyzed during the current study are not publicly available due to the containing information that could compromise research participant privacy and consent but are available from the corresponding author upon reasonable request.
